# Structural characteristics of a neutral *Glycyrrhiza uralensis* polysaccharide and its fermentation properties on the gut microbiota of immunocompromised rats *in vivo* and *vitro*

**DOI:** 10.3389/fnut.2025.1651015

**Published:** 2025-10-07

**Authors:** Jie Sun, Yi-Xuan Wu, Xin-Li Li, Wen-Jie Xie, Qing-Ping Xiong, Chang-Xing Jiang, Shu-Wan Tang, Guo-Ping Peng, Yun-Feng Zheng

**Affiliations:** ^1^Department of Pharmacy, Nanjing University of Chinese Medicine, Nanjing, China; ^2^National Key Laboratory on Technologies for Chinese Medicine Pharmaceutical Process Control and Intelligent Manufacture, Nanjing, China; ^3^Jiangsu Province Engineering Research Center of Classical Prescription, Nanjing University of Chinese Medicine, Nanjing, China; ^4^Jiangsu Key Laboratory of Regional Resource Exploitation and Medicinal Research, Huaiyin Institute of Technology, Huai’an, China; ^5^Jiangsu Collaborative Innovation Center of Chinese Medicinal Resources Industrialization, Nanjing University of Chinese Medicine, Nanjing, China

**Keywords:** *Glycyrrhiza uralensis*, neutral polysaccharide, structural characterisation, gut microbiota, fermentation

## Abstract

Glycyrrhiza polysaccharides (GPs) exhibit notable physiological activity; however, their structure is not well understood. The novel neutral polysaccharide fraction GP-1 was isolated from the roots and rhizomes of *Glycyrrhiza uralensis* Fisch. The fermentation properties of GP-1 were investigated *in vitro* and *in vivo* in immunocompromised rats. The molecular weight (Mw) of GP-1 is 7.6 kDa. The analysis of the monosaccharide composition indicated that GP-1 is a glucan. Nuclear magnetic resonance spectroscopy and methylation analyses revealed that GP-1 comprised a glucan main chain linked by α-D-Glc*p*-(1 → 4) bonds, with β-D-Glc*p*-(1 → 6)-α-D-Glc*p*-(1 → side branches at the C-6 position of 1,4,6-Glc and β-D-Glc*p*-(1 → side branches at the C-3 position of 1,3,4-Glc. Staining with Congo red confirmed the presence of a triple-helix structure. Scanning electron microscopy revealed that GP-1 has granular morphology. Pharmacological studies showed that GPs and GP-1 modulated the balance of the gut microbiota and influenced the production of short-chain fatty acids. In addition, the *in vivo* fermentation of GP and the *in vitro* fermentation of GP-1 promoted the growth of certain probiotics, particularly *Lactobacillus* and *Dubosiella*. These results underscore the structural characteristics of GP-1 and its potential as a prebiotic agent.

## 1 Introduction

Glycyrrhiza is sourced from the roots and rhizomes of Glycyrrhiza uralensis (*G. uralensis*) Fisch and is extensively utilized in food and medicine (1). Contemporary studies have shown that glycyrrhiza possesses multiple advantageous pharmacological properties, such as antioxidant, anti-inflammatory, antiviral, anti-allergic, anti-ulcer, and anti-diabetic effects (2). The advantageous characteristics are due to glycyrrhiza’s abundant chemical makeup, which comprises saponins, flavonoids, polysaccharides, coumarins, alkaloids, volatile oils, amino acids, and trace elements (1, 3–5). While the small molecular elements of glycyrrhiza, including glycyrrhizic acid, glycyrrhetinic acid, liquiritin, and isoliquiritin, have been thoroughly researched (6–8), glycyrrhizic polysaccharides are still mostly unexamined.

Recently, glycyrrhiza polysaccharides (GPs) have attracted considerable interest, with substantial evidence supporting their antioxidant, antibacterial, antiviral and anti-inflammatory properties, as well as their ability to modulate the gut microbiota (9, 10). Recent research has demonstrated that GPs can also exhibit biomimetic anti-acne effect when applied topically (11). The structure of GPs was examined in more detail by researchers. Mutaillifu et al. managed to isolate a water-soluble polysaccharide that is primarily glucose-based from *Glycyrrhiza glabra* (12). Pan et al. isolated a homogeneous polysaccharide with a molecular weight of 1.96 × 10^3^ kDa from *Glycyrrhiza inflata* Batalin (13). Wu et al. isolated an acidic polysaccharide with a molecular weight of 26.4 kDa from *G. uralensis* (14). The structure of the three polysaccharides mentioned above has been systematically characterized, and their antioxidant, anti-α-glucosidase, and immunomodulatory activities have been preliminarily evaluated. Aipire et al. isolated three polysaccharide components (GUPS-i, GUPS-ii and GUPS-iii) from *G. uralensis* with molecular weights of 1.06, 29.1 and 14.9 kDa, respectively (15). The preliminary analysis indicated that GUPS-i is a neutral polysaccharide, whereas GUPS-ii and GUPS-iii are acidic polysaccharides. More studies are needed to clarify the exact glycosidic bond connections and other pertinent information. Among the three medicinal glycyrrhiza species, *G. uralensis* is the most commonly used. Thorough studies of its polysaccharide structure will lay an essential groundwork for the standardization and full use of glycyrrhiza resources.

The body’s metabolism, immune system, and mental state are significantly affected by gut health (16, 17). Polysaccharides are promising prebiotics. Since the human gastrointestinal system does not have active enzymes to break down carbohydrates (18), certain polysaccharides can reach the colon undigested, where they are fermented and utilized by the microbial community there. This process helps sustain equilibrium in the gut microecology and promotes microbial diversity (19, 20). During digestion in the gut, polysaccharides are broken down by the colonic microbes to form short-chain fatty acids (SCFAs), prebiotics, and other advantageous substances, which in turn affect the gut microbiota and support host health (21, 22). Our previous research indicated that the significant increase in water-soluble polysaccharides following the honey-roasting of glycyrrhiza may be one of the primary factors through which glycyrrhiza enhances immune regulation via the gut microbiota and SCFA pathway (23). Nonetheless, the effects of GPs on the gut microbiota and their metabolites in rats with weakened immune systems have not been fully studied, necessitating additional research.

In this study, diethylaminoethyl (DEAE) anion exchange chromatography was employed to isolate and purify a novel neutral polysaccharide fraction (GP-1) from *G. uralensis* Fisch. Building on existing research on GPs, the primary objectives of this study were (1) to conduct a comprehensive structural analysis of GP-1, focussing on its molecular weight, monosaccharide composition and glycosidic linkage; (2) to investigate the fermentation properties of crude GP in immunocompromised rats; and (3) to examine the fermentation characteristics of purified GP-1 *in vitro*.

## 2 Materials and methods

### 2.1 Materials and reagents

Glycyrrhiza tablets were purchased from Shengshi Hyakuso Co. (Tianjin, China) and identified by Associate Professor Hui Yan as the roots and rhizomes of *G. uralensis* Fisch. DEAE-52 cellulose was purchased from Solarbio (Beijing, China). A series of standard dextrans with known molecular weights (5,000, 11,600, 23,800, 48,600, 80,900, 148,000, 273,000, 409,800 and 667,800 Da) were purchased from Aladdin Reagent Co., Ltd. (Shanghai, China). 3-nitrophenylhydrazine hydrochloride (3-NPH) (>98%) and N-(3-dimethylaminopropyl)-N-ethylcarbodiimide hydrochloride (EDC) (>98%) were purchased from Sigma-Aldrich (St. Louis, MO, USA). SCFAs (acetate, propionate, butyrate, 2-methylbutyrate, isobutyrate, valerate and isovalerate) and the internal standard (IS, d3-hexanoic acid-d3) were also purchased from Sigma-Aldrich (St. Louis, MO, USA). All other chemicals and reagents used were of analytical grade.

### 2.2 Extraction and purification of GP-1

Glycyrrhiza slices underwent two extractions with water in a 1:10 ratio (slices to water). The resulting filtrates were merged and processed through a D101 macroporous resin column. The effluent was gathered, concentrated under low pressure, and adjusted to a 65% alcohol level with ethanol to create a precipitate. Following protein removal via the Sevage method, the samples were freeze-dried to yield crude GPs.

The raw GPs were dissolved in ultrapure water and then separated with a DEAE-52 cellulose chromatography column (24, 25). Polysaccharides were eluted from the column using ultrapure water and NaCl solutions (0.1, 0.3, and 0.5 M) as the mobile phases, with a flow rate of 1 mL/min, collecting 10 mL per tube. The phenol-sulfuric acid method was used for detection. Polysaccharide-containing fractions were pooled, concentrated, and dialyzed with a membrane having a molecular weight cut-off of 3,000 Da. The dialysis process involved running tap water for 15 h, followed by distilled water for 20 h, with the distilled water being replaced five times. The concentrated solution in the dialysis bag was then collected and freeze-dried to produce uniform GP-1.

### 2.3 Structural characterisation

#### 2.3.1 Quantification of sugar, protein, and sulphate

The phenol–sulphuric acid method was used to measure the total sugar content in GP and GP-1, using glucose as the standard reference (24). The Bradford method was employed to measure protein content, using bovine serum albumin as the standard (19). The m-hydroxydiphenyl method, with galacturonic acid as the standard, was used to measure the glucuronide content of GP-1 (25).

#### 2.3.2 Determination of homogeneity and average molecular weight

High-performance gel permeation chromatography (HPGPC) was used to determine the homogeneity and molecular weight (Mw) of GP-1 (10, 26). The chromatographic setup included a sequence of BRT105-104-102 gel columns, each measuring 8 mm by 300 mm. The mobile phase used was a 0.05 M NaCl solution, with a flow rate of 0.6 mL per minute and a column temperature maintained at 40 °C. A 20 μL sample injection volume was used, and detection was carried out with a differential detection technique. The relative molecular weights of GP-1 (5,000, 11,600, 23,800, 48,600, 80,900, 148,000, 273,000, 409,800, and 667,800) were calculated using the standard dextran curve. *2.3.3. Analysis of monosaccharide composition.*

To verify the monosaccharide composition of GP-1, high-performance liquid chromatography (HPLC) was utilized, with precolumn derivatization using 1-phenyl-3-methyl-5-pyrazolone (PMP) as outlined in an earlier study Sun et al. (27). Around 2 mg of polysaccharide was fully broken down with 1.0 mL of 2 M trifluoroacetic acid (TFA) at 100 °C over a period of 10 h. Following the removal of TFA using vacuum rotary evaporation, the dried residue was dissolved in 300 μL of distilled water and underwent PMP derivatisation. The derivatised samples underwent three extractions with 600 μL of chloroform and were subsequently analyzed using an HPLC system featuring a Hedra C18 column (5 μm, 4.6 mm × 250 mm) linked to an Agilent 1,100 system (Agilent, Santa Clara, CA, USA). Detailed experimental procedures are provided in Supplementary material S2.

#### 2.3.3 Methylation and gas chromatography–mass spectrometry (GC–MS) analyses

To identify the glycosidic linkages in GP-1, we used a methylation analysis method that was slightly modified from a reported procedure (28, 29). We transferred 3 mg of GP-1 to a glass reaction vial and added 1 mL of anhydrous dimethylsulfoxide (DMSO). Methylating reagent A (anhydrous alkaline solution) was rapidly added, and the vial was sealed. The mixture was then subjected to ultrasonic dissolution, followed by the addition of methylating reagent B, an iodomethane solution. The reaction was carried out at 30 °C in a magnetically stirred water bath for a duration of 60 min. To terminate the methylation reaction, 2 mL of ultrapure water was added. The methylated polysaccharide was subjected to hydrolysis using 1 mL of 2 M trifluoroacetic acid (TFA) and incubated for 90 min, after which it was dried. The resulting residue was dissolved in 2 mL of ultrapure water and subsequently reduced with 60 mg of sodium borohydride for a duration of 8 h. The reaction mixture was neutralized with ice-cold acetic acid, and the product was concentrated through rotary evaporation prior to being dried at 101 °C. Subsequently, 1 mL of acetic anhydride was added, and the reaction was carried out at 100 °C for 1 h, followed by a cooling phase. Following the addition of 3 mL of toluene, the mixture underwent concentration under reduced pressure, a procedure that was repeated four to five times to eliminate excess acetic anhydride. Subsequently, the acetylated product was dissolved in 3 mL of dichloromethane (CH2Cl2), transferred to a separatory funnel, and subjected to vigorous agitation with a minimal volume of distilled water. The upper aqueous phase was discarded, and this extraction process was repeated four times. The dichloromethane layer was then dried using an appropriate amount of anhydrous sodium sulfate, and its volume was adjusted to 10 mL. The acetate esters of the methylated sugar alcohols were analysed using a GC–MS system (GC–MS-QP2010).

GC–MS conditions: RXI-5 SIL MS column (30 mm × 0.25 mm, 0.25 μm); temperature program began at 120 °C, ramped at 3 °C/min up to 250 °C, where it was held for 5 min; injection port and detector temperatures were set to 250 °C and helium was used as the carrier gas at a flow rate of 1 mL/min.

#### 2.3.4 Fourier transform infrared (FT-IR) analysis

GP-1 (1 mg) was finely ground with dried KBr and pressed into a pellet. The IR spectrum was recorded using a Nicolet FT-IR spectrometer (Nicolet IR100, Thermo Scientific, USA) in the wavelength range of 4,000–400 cm^−1^ (30).

#### 2.3.5 Nuclear magnetic resonance (NMR) spectroscopy

We dissolved 20 mg of GP-1 in 0.5 mL of deuterium oxide and subjected it to freeze-drying. This procedure was repeated three times to guarantee adequate exchange of labile hydrogen. The GP-1 sample was then dissolved in 0.5 mL of heavy water and analysed using 1D and 2D NMR spectroscopy using a 600 MHz NMR (AAVANCE NEO 600 M, Bruker, Germany).

#### 2.3.6 Congo red analysis

The Congo red staining technique was employed to characterize the triple-helix structure of GP-1 (31). The polysaccharides were dissolved in distilled water at a concentration of 1 mg/mL, together with a 100 μM Congo red solution. The polysaccharide solution was combined in equal parts with Congo red reagent, and then the NaOH concentration of the mixture was adjusted using a 1 M NaOH solution within the range of 0–0.5 M. Spectral scanning in the 400–900 nm range was used to find the maximum absorption wavelength of the samples at various NaOH concentrations (32).

#### 2.3.7 Scanning electron microscopy (SEM)

SEM was used to examine the morphology of GP-1. The sample was placed on a copper stub and covered with a thin gold layer through sputter coating before analysis at 2.0 kV (33).

### 2.4 *In vivo* fermentation of GP in rats

#### 2.4.1 Animals and experimental design

Male Wistar rats, weighing between 180 and 220 grams and aged eight weeks, were obtained from Shanghai Shilaike Laboratory Animal Co., Ltd. in Shanghai, China, and kept at the Nanjing University of Chinese Medicine’s animal facility. The animals were kept at room temperature (20 °C–25 °C) with constant relative humidity under a 12-h light/dark cycle. Prior to the experiment, the rats were allowed to adjust to the lab environment for a week, with unlimited access to food and water. All experimental protocols and procedures were approved by the Experimental Animal Ethics Committee of the Nanjing University of Chinese Medicine (ethics approval number: 202207A019). Furthermore, the animal studies followed the guidelines set by the National Research Council’s *Guide for the Care and Use of Laboratory Animals*.

The rats were randomly divided into control (CON), model (MOD), low-dose GP (0.84 g/kg) and high-dose GP (3.36 g/kg) groups. All rats except those in the CON group were placed in a glass cylinder (diameter = 31 cm and depth = 48 cm) maintained at 37 °C ± 1 °C (the tank diameter and water depth were adjusted visually according to the size of the rat) and forced to swim until exhaustion. They were given 75 g/kg body weight of feed every other day to induce an immunocompromised state (23). After two weeks, the rats in the CON group were given sterile physiological saline through intragastric feeding once a day for two weeks. Meanwhile, the rats in the other groups were administered oral GP daily for the same duration.

The rats were anesthetized with sodium pentobarbital through an intraperitoneal injection 24 h after the last dose. Blood was drawn from the abdominal aorta, and the rats were euthanized. Intestinal tissues and feces were gathered.

#### 2.4.2 Histopathological study

The ileum tissue was preserved in 10% neutral formalin, set in paraffin, and then stained using haematoxylin and eosin (H&E). For histopathological analysis, microscopic images were taken with a 100 × objective lens to examine the length and spacing of intestinal villi.

#### 2.4.3 Enzyme-linked immunosorbent assay (ELISA)

Blood samples were centrifuged at 4 °C for 10 min at 3,500 × *g* to isolate the sera. The levels of cytokines (IL-2, IL-4, TNF-α and IFN-*γ*) and immunoglobulins (IgA and IgG) in the sera were quantified using an ELISA kit provided by Nanjing Lapuda Biotechnology Co., Ltd. (China).

### 2.5 *In vitro* fermentation of GP by rat faeces

An *in vitro* fermentation of rat fecal microbiota was conducted to investigate the potential biological activity of GP-1, resulting in only minor alterations. In short, a fundamental fermentation medium (1.0 L) was created by dissolving 47.5 g of minced meat carbohydrate broth in 1 L of deionized water. The mixture was heated, portioned, and enhanced with a suitable quantity of minced beef. The blend was subsequently sterilized in a high-pressure autoclave at 121 °C for 15 min and then cooled for future use. Rats’ fecal samples from the experimental group were combined with a sterile phosphate buffer solution (0.1 M, pH 7.0) in a 1:10 (v/v) ratio under anaerobic conditions. The blend was homogenized, passed through sterile gauze, and diluted by a factor of 100. GP-1 at concentrations of 2 and 10 mg/mL was combined with 11.7 mL of the base culture medium and 1.3 mL of the fecal bacteria suspension. In the CON group, just the basic culture medium was utilized. Afterward, all samples were incubated at 37 °C for 12 h and then collected. Post-fermentation, the samples were spun at 8,000 rpm for 20 min to divide the supernatant from the precipitate. The isolated parts were then frozen at −80 °C for later use.

### 2.6 Analysis of gut microbiota composition

Fresh faecal samples and *in vitro* fermentation products were analysed according to an established method (23).

### 2.7 Targeted metabolite analysis

Fresh fecal samples and products from *in vitro* fermentation were kept at −80 °C. Short-chain fatty acids (SCFAs) such as acetate, propionate, butyrate, 2-methyl-butyrate, isobutyrate, valerate, and isovalerate were extracted and measured using a documented technique. Briefly, SCFAs were converted into 3-nitrophenylhydrazone using 3-NPH, and deuterated analogues like hexanoic acid-d3 served as internal standards. Liquid chromatography–tandem mass spectrometry (LC–MS/MS) was used to measure the concentration of faecal SCFAs, with detailed procedures available in Supplementary material S3.

### 2.8 Statistical analysis

Data were expressed as mean ± standard deviation (SD). The repeated measures data were analyzed using one-way ANOVA and Sidak *post hoc* test. *p* value <0.05 was regarded as statistically significant. All statistical figures were built using GraphPad statistical software (GraphPad Software Inc., Chicago, IL, USA).

## 3 Results and discussion

### 3.1 Purification and chemical composition of GP-1

**Figure 1:**
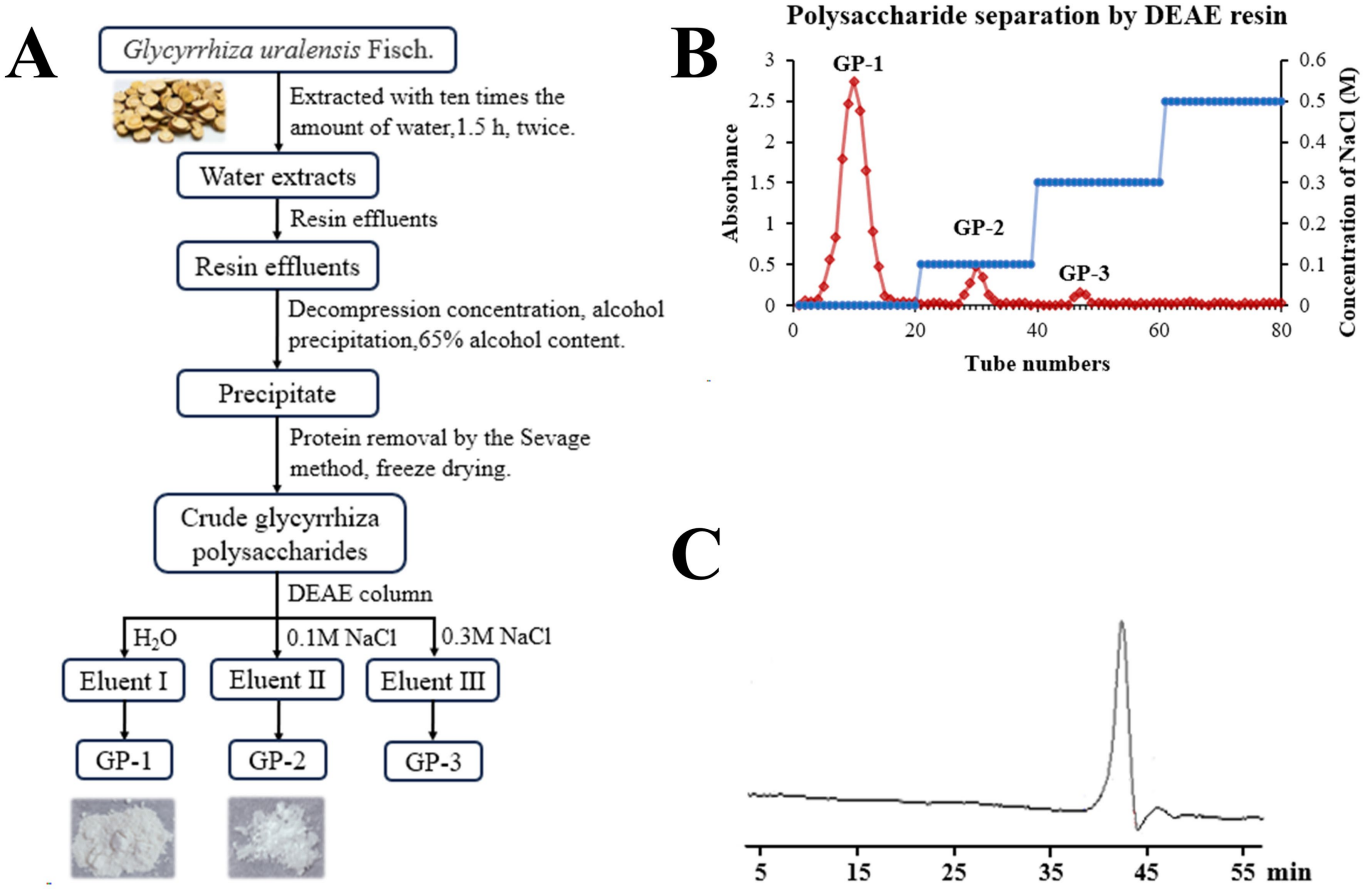
Isolation and molecular weight determination of neutral polysaccharides in *Glycyrrhiza uralensis* (*G. uralensis*). **(A)** Extraction and purification scheme for GP-1. **(B)** Diethylaminoethyl (DEAE) elution profile. **(C)** High-performance gel permeation chromatography (HPGPC) results for GP-1.

Crude polysaccharides were extracted from glycyrrhiza using water extraction, large-pore resin impurity removal and concentrated alcohol precipitation, followed by treatment with the Sevage reagent. This process yielded 6.3% crude polysaccharides. Further, DEAE anion exchange chromatography was employed for separation and purification, resulting in the isolation of three major GP components: GP-1, GP-2 and GP-3 in 3.4, 0.7 and 0.2% yields, respectively (Figures 1A,B). Because of the low yields of GP-2 and GP-3 after purification, only GP-1 was studied in subsequent experiments.

The total carbohydrate content of GP-1 was 96.89%, and it contained 1.80% uronic acid and 0.68% protein.

### 3.2 Structural characterisation of GP-1

#### 3.2.1 Mw and monosaccharide composition of GP-1

**Figure 2:**
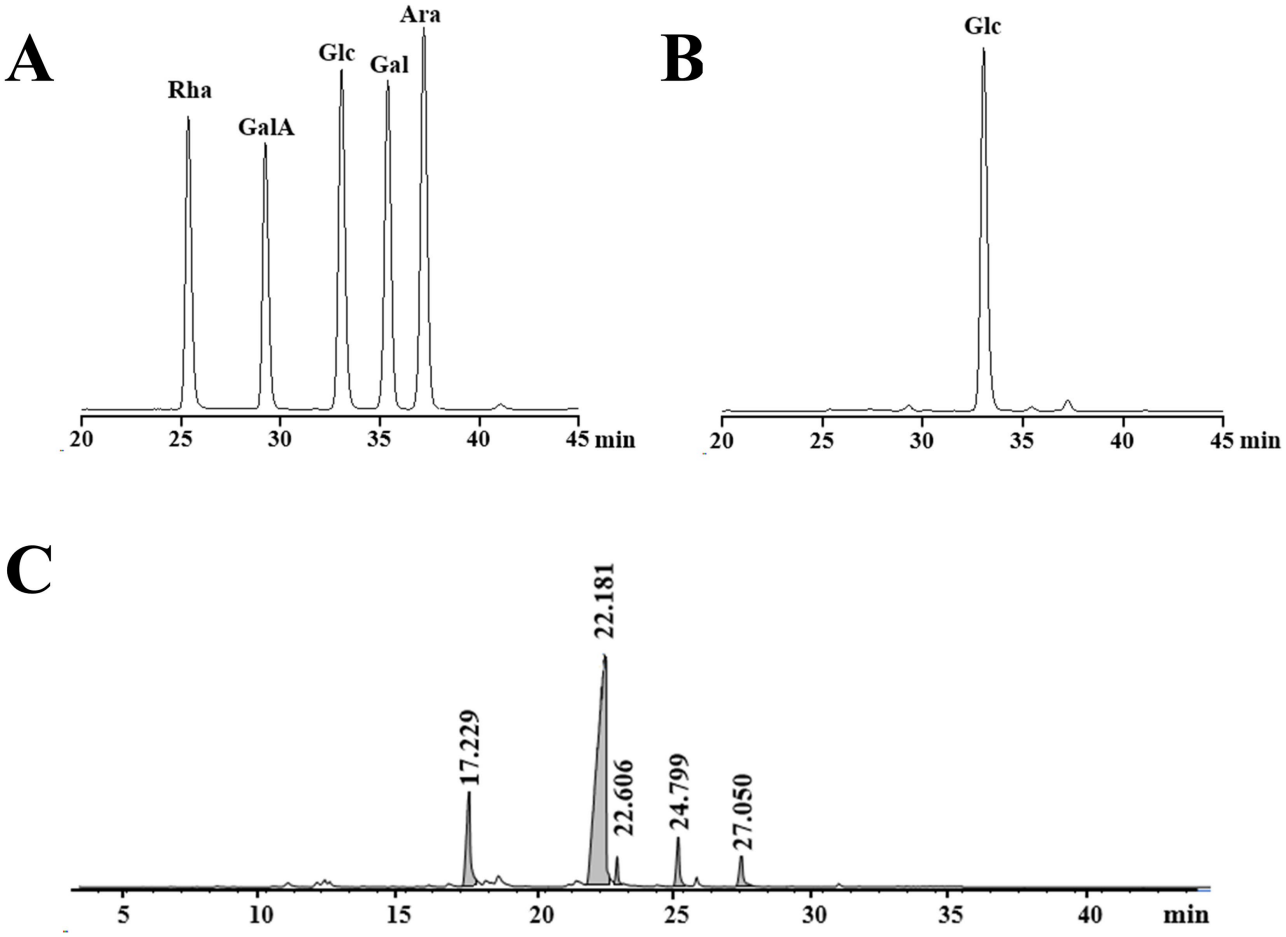
High-performance liquid chromatography (HPLC) and gas chromatography–mass spectrometry (GC–MS) profiles of GP-1. **(A)** HPLC profile of monosaccharide standards. **(B)** HPLC profile following complete acid hydrolysis of GP-1. **(C)** GC–MS profile after methylation.

The GPC chromatogram exhibited a relatively uniform and symmetric peak, indicating that GP-1 is a highly pure and homogenous polysaccharide (Figure 1C). The GPC analysis revealed that the Mw of GP-1 was 7.6 kDa. Its relatively low molecular weight endows it with superior water solubility and dispersion performance. Comparison with the monosaccharide standard confirmed that GP-1 is composed of glucose (Figures 2A,B). Neutral polysaccharides from *G. uralensis* are primarily composed of glucans, as described in a previous study (12). However, significant differences in Mw were observed, which can be attributed to variations in the raw materials and processing methods used.

#### 3.2.2 Methylation analysis

Methylation analysis is widely used to determine polysaccharide structure. The method involves converting the individual constituent sugars in polysaccharides into partially methylated alditol acetates, which are then analysed and quantified by GC–MS. The proposed method enables the identification of attachment sites and molar ratios of different sugar residues. The results of the methylation analysis are presented in Table 1 and Figure 2C. Based on the GC–MS spectral database (Agilent), retention times of each peak, main characteristic fragments and published spectral analysis results (30, 34), we identified five different glycosidic bonds in GP-1, T-Glc*p*-(1→, →4)-Glc*p*-(1→, →6-Glc*p*-(1→, →3,4)-Glc*p*-(1 → and →4,6)-Glc*p*- (1→, with molar ratio of 0.150:0.733: 0.024:0.051:0.041.

**Table 1:** Results of the methylation analysis of GP-1.

RT	Methylated sugar	Mass fragments (*m/z*)	Molar ratio	Type of linkage
17.299	2,3,4,6-Me4-Glc*p*	43, 71, 87, 101, 117, 129, 145, 161, 205	0.150	Glc*p*-(1→
22.181	2,3,6-Me3-Glc*p*	43, 87, 99, 101, 113, 117, 129, 131, 161, 173, 233	0.733	→4)-Glc*p*-(1→
22.606	2,3,4-Me3-Glc*p*	43, 87, 99, 101, 117, 129, 161, 189, 233	0.024	→6-Glc*p*-(1→
24.799	2,6-Me2-Glc*p*	43, 87, 97, 117, 159, 185	0.051	→3,4)-Glc*p*-(1→
27.050	2,3-Me2-Glc*p*	43, 71, 85, 87, 99, 101, 117, 127, 159, 161, 201	0.041	→4,6)-Glc*p*-(1→

#### 3.2.3 FT-IR spectrum analysis

**Figure 3:**
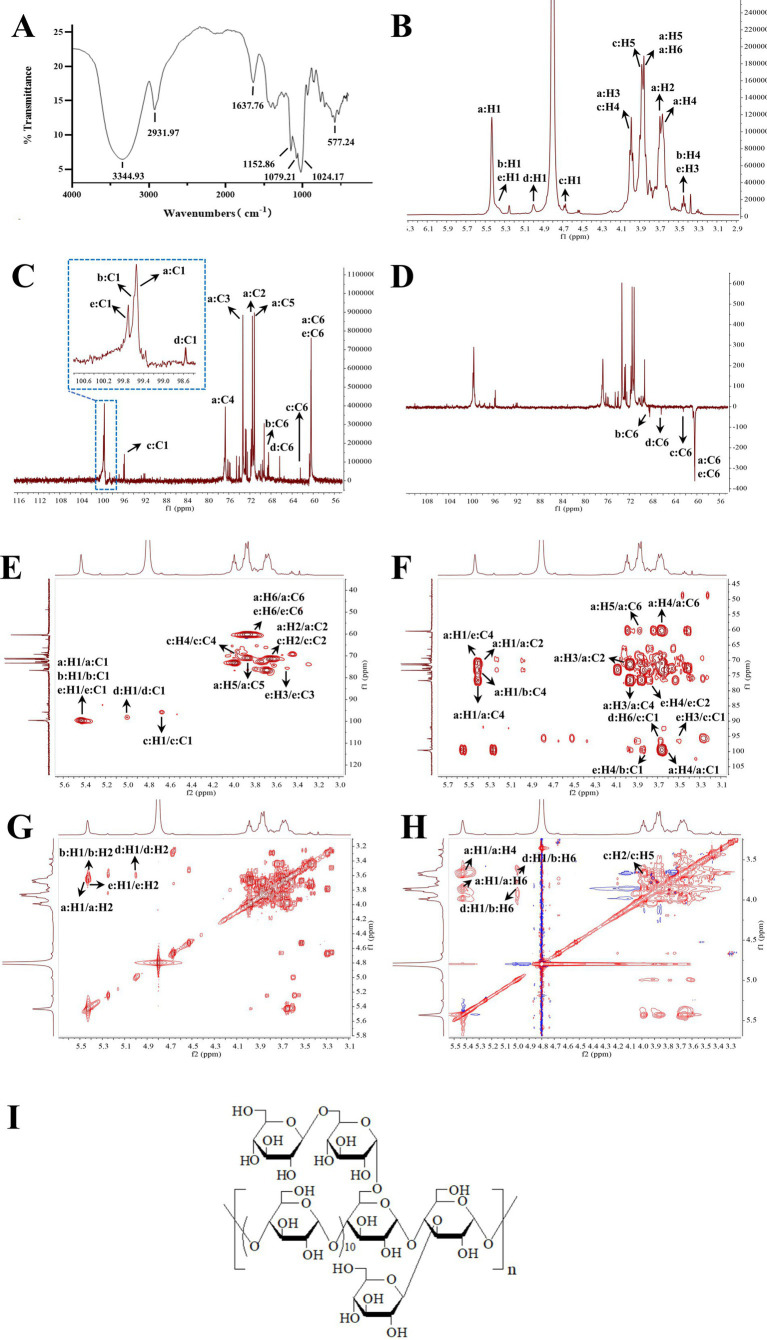
Infrared (IR) and nuclear magnetic resonance (NMR) spectra of GP-1. **(A)** IR spectrum of GP-1; **(B)** ^1^H NMR spectrum of GP-1; **(C)** ^13^C NMR spectrum of GP-1; **(D)** Dept-135 NMR spectrum of GP-1; **(E)** HSQC spectrum of GP-1; **(F)** HMBC spectrum of GP-1; **(G)** COSY spectra of GP-1; **(H)** NOESY spectra of GP-1; **(I)** Structure of GP-1.

As shown in Figure 3A, the FT-IR spectrum of GP-1 exhibited a broad characteristic absorption peak in the range of 3,500–3,000 cm − 1, which can be attributed to the stretching vibration of the O–H bonds in polysaccharides (35). The absorption peak at 2931.97 cm − 1 corresponded to the stretching vibration of the C–H bonds in polysaccharides (36). The absorption peak at approximately 1,100 cm − 1 can be attributed to the stretching vibration of the C–O bonds, indicating the presence of functional groups in the polysaccharide (37). The peak at 1637.76 cm − 1 indicated the presence of free hydroxyl groups in bound water (31). Additionally, the absorption peaks at 1024.17, 1079.23 and 1152.86 cm − 1 indicated the presence of pyranose sugars in the polysaccharide composition of GP-1 (38).

#### 3.2.4 NMR analysis

To determine the linkage sequence of each sugar residue in GP-1, we performed 1D and 2D NMR analyses. The proton signals in the ^1^H NMR spectrum were primarily concentrated between *δ*_H_ 3.0 and 5.5 ppm, with the signals corresponding to the sugar rings appearing in the range of *δ*_H_ 3.4–4.0 ppm (Figure 3B). The signal peaks of the main terminal protons at *δ*_H_ 5.43, 5.37, 5.36, 5.00 and 4.53 were predominantly distributed in the range of *δ*_H_ 4.3–5.5 ppm. In the ^13^C NMR and heteronuclear single quantum coherence (Figures 3C,D) spectra, the carbon signals at *δ*_C_ 99.55, 99.59, 99.71, 98.60 and 96.75 ppm were mainly located in the range of *δ*_C_ 95–105 ppm. The signal peaks at *δ*_C_ 71.51, 73.32, 76.66, 71.15, 60.45, 71.46, 74.11, 73.99, 72.69, 68.47, 71.71, 73.32, 69.30, 72.44, 62.44, 72.44, 74.52, 70.33, 72.85, 66.36, 76.18, 75.74, 70.16, 75.93 and 60.66 ppm were primarily distributed in the range of *δ*_C_ 60–85 ppm.

Based on the ^13^C NMR and HSQC spectra (Figure 3E), GP-1 primarily exhibited five continuous oxygenated carbon signals at *δ*_C_ 71.51 (CH), 73.32 (CH), 76.66 (CH), 71.15 (CH) and 60.45 (CH_2_) and a terminal carbon signal at 99.55 ppm (CH). The anomeric hydrogen signal of β-glycosidically bonded configurations was mainly distributed in the range of δ 4.4–4.8 ppm, whereas that of α-glycosidically bonded configurations primarily appeared in the range of δ 4.8–5.8 ppm (31). The chemical shift of H-1 at 5.43 indicates the presence of an α-glycosidic bond. The heteronuclear multiple bond correlation (HMBC) spectra revealed correlation signals between H-1 and C-4, as well as between H-4 and C-1, indicating that the glycosidic bond in the neutral polysaccharide was linked at the α-(1,4) position. By correlating the results of methylation with those of relevant literature (12, 13), 1,4-Glc was identified as the GP-1 main chain, and the primary connection of the main chain was a: →4)-α-D-Glc*p*-(1→. The glycosidic bond signalling of GP-1, considering its spatial structure, was further corroborated by correlating the methylation data and HSQC, HMBC, correlation spectroscopy (COSY) and nuclear Overhauser effect spectroscopy (NOESY) spectra, as well as extensive literature reports (12, 30, 31, 34). These findings are summarized in Table 2 and Figures 3E–H.

**Table 2:** GP-1 glycosidic bond signaling attribution.

Glycosyl residues	H1/C1	H2/C2	H3/C3	H4/C4	H5/C5	H6a, b/C6
a: →4)-α-D-Glc*p*-(1→	5.43	3.65	3.97	3.63	3.82	3.83
	99.55	71.51	73.32	76.66	71.15	60.45
b: →4, 6)-α-D-Glc*p*-(1→	5.37	3.57	3.69	3.42	3.62	3.86, 3.56
	99.59	71.46	74.11	73.99	72.69	68.47
c: β-D-Glc*p*-1→	4.53	3.65	3.73	3.99	3.84	3.74, 3.99
	96.75	71.71	73.32	69.30	72.44	62.44
d: →6)-α-D-Glc*p*-(1→	5.00	3.56	3.69	3.43	3.86	3.65, 3.89
	98.60	72.44	74.52	70.33	72.85	66.36
e: →3, 4)-α-D-Glc*p*-(1→	5.36	3.67	3.40	3.76	3.60	3.84
	99.71	76.18	75.74	70.16	75.93	60.66

All the glycosidic bonds were in the α-conformation except for c: β-D-Glcp-1→, which exhibited an isohead carbon signal at 4.53 and adopted a β-conformation. In the HMBC spectrum, a correlation peak was observed between C-4 of the b: →4,6)-α-D-Glc*p*-(1 → glycosidic bond and H-1 of the a: →4)-α-D-Glc*p*-(1 → glycosidic bond, indicating the presence of the →4)-α-D-Glc*p*-(1 → 4,6)-α-D- Glc*p*-(1 → glycosidic bond. Similarly, a correlation peak was observed between C-4 of the e: →3,4)-α-D-Glc*p*-(1 → glycosidic bond and H-1 of the a: →4)-α-D- Glc*p*-(1 → glycosidic bond, indicating the presence of the →4)-α-D-Glc*p*-(1 → 3,4)- α-D-Glc*p*-(1 → glycosidic bond. Furthermore, a correlation peak was detected between C-1 of the c: β-D-Glc*p*-1 → glycosidic bond and H-6 of the d: →6)-α-D- Glc*p*-(1 → glycosidic bond, indicating the presence of the β-D-Glc*p*-(1 → 6)-α-D- Glc*p*-(1 → glycosidic bond, as shown in Figure 3F.

According to the NOESY spectrum, a correlation peak was observed between H-1 of the b: →4,6)-α-D-Glc*p*-(1 → glycosidic bond and H-4 of the e: →3,4)-α-D-Glc*p*-(1 → glycosidic bond, indicating the presence of the →4,6)-α-D-Glc*p*-(1 → 3,4)-α-D- Glc*p*-(1 → glycosidic bond. Another correlation peak was detected between H-3 of the e: →3,4)-α-D-Glc*p*-(1 → glycosidic bond and H-1 of the c: β-D-Glc*p*-1 → glycosidic bond, indicating the presence of the β-D-Glc*p*-1 → 3,4)-α-D-Glc*p*-(1 → glycosidic bond. In addition, the correlation signal between H-6 of the b: →4,6)-α-D-Glc*p*-(1 → glycosidic bond and H-1 of the d: →6)-α-D-Glc*p*-(1 → glycosidic bond confirmed the presence of the →6)-α-D-Glc*p*-(1 → 4,6)-α-D -Glc*p*-(1 → glycosidic bond, as shown in Figure 3H.

Based on these results, we propose that the primary glycosidic bond structure of GP-1 is characterized by a main chain linkage of →4)-α-D-Glc*p*-(1→, with β-D-Glc*p*-(1 → 6)-α-D-Glc*p*-(1 → substitution at the C-6 position of 1,4,6-Glc and β-D-Glc*p*-(1 → substitutions at the C-3 position of 1,3,4-Glc, as shown in Figure 3I.

α-(1 → 4) Linkage is a typical connection method for certain degradable energy-storing polysaccharides. This type of linkage is more susceptible to enzymatic hydrolysis, which generates oligosaccharide fragments with β-glycosidic bonds that are more readily metabolized by microorganisms. The presence of β-(1 → 6)-side chains enhances the molecular’s three-dimensional conformational flexibility and high branching, enabling it to form a larger contact area when binding to receptors. This increases the likelihood of multi-site interactions, thereby strengthening its binding affinity with immune-related active centers. Additionally, the C-3 β-Glcp side chain alters the stereochemical environment of the main chain, facilitating the formation of unique spatial conformations that improve specific biological activities. These mechanisms contribute to functions such as inhibiting pathogenic viral infections or enhancing the efficiency of host defense signaling transduction (39, 40).

#### 3.2.5 Congo red analysis

**Figure 4:**
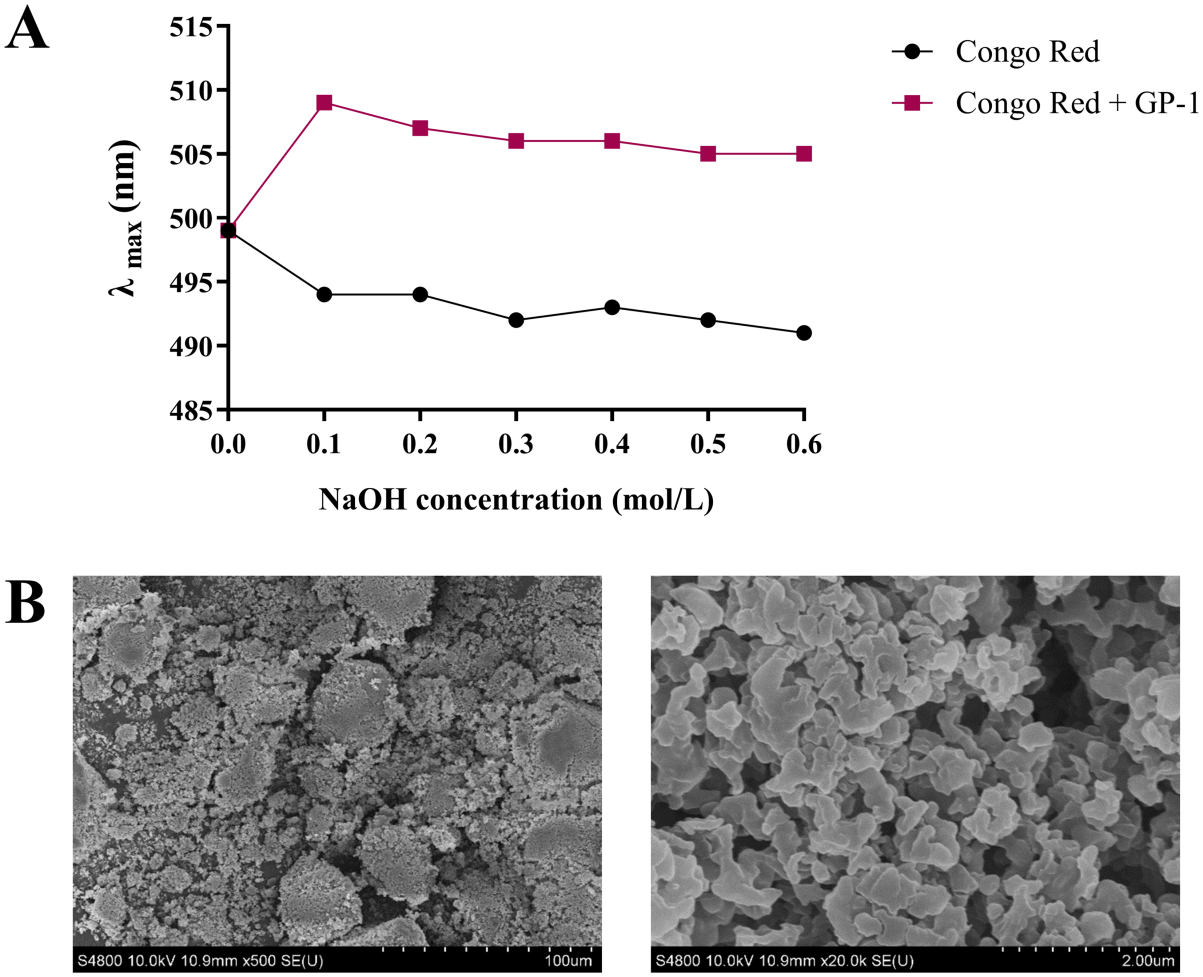
Scanning electron microscopy (SEM) and Congo red analyses of GP-1. **(A)** Maximum absorption wavelengths of Congo red-stained GP-1 at various NaOH concentrations. **(B)** SEM image of GP-1 (500 × and 20,000×).

The results of the Congo red experiment are shown in Figure 4A. At low NaOH concentrations, the maximum absorption wavelength of the Congo red-stained polysaccharide solution exhibited a distinct red shift. However, when the NaOH concentration exceeded 0.1 mol/L, a decreasing trend was observed at the maximum absorption wavelength, with no indication of a stable phase throughout the process. The maximum absorption wavelength of the Congo red-polysaccharide solution may not have decreased with increasing NaOH concentration if light protection was not applied during the experiment. As the NaOH concentration increased, the triple-helix structure of the Congo red-GP-1 complex became disrupted, reducing the maximum absorption wavelength. Literature indicates that at low alkali concentrations, a pronounced red shift is typically observed, which can be attributed to the presence of the triple helix, even in the absence of a stabilization zone in the absorption curve (31, 41). Based on these observations, we hypothesized that GP-1 possesses a triple-helix structure.

#### 3.2.6 Ultrastructural properties

SEM is a qualitative technique for observing the surface morphology of polysaccharides. As shown in Figure 4B, GP-1 exhibited a smooth reticulated structure composed of many spherical particles stacked on one another, indicating strong intermolecular interactions and tight binding within the sample (30). This result was consistent with its low molecular weight (15).

### 3.3 Enhancement of the immunomodulatory effect of GP in immunocompro- mised rats

**Figure 5:**
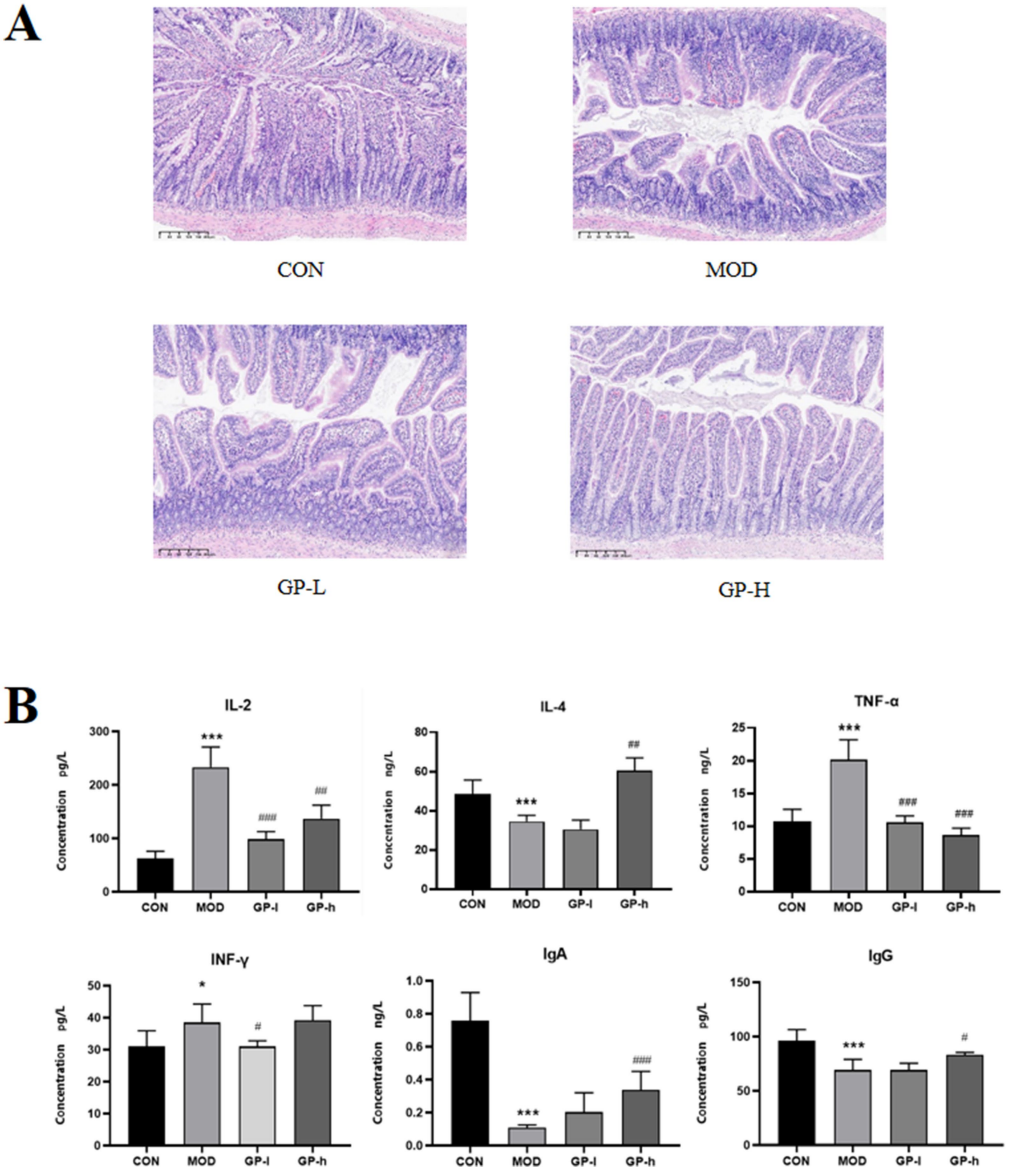
Effects of glycyrrhiza polysaccharides (GPs) on immune function in immunocompromised rats (*n* = 6). A Effects on small intestinal villi. B: Effects on cytokine levels. CON: control group, MOD, model group; GP-L, low-dose GP group; GP-H, high-dose GP group. The values are expressed as means ± SD. The repeated measures data were analyzed using one-way ANOVA and Sidak *post hoc* test (vs. CON, *p < 0.05, ***p < 0.001; vs. MOD, # *p* < 0.05, ##*p* < 0.01, ###*p* < 0.001).

To evaluate the immunomodulatory effects of GP, we assessed the morphological structure of the intestine and quantitatively measured the levels of immune-related cytokines (IL-2, IL-4, TNF-α and IFN-*γ*) and immunoglobulins (IgA and IgG) in the serum. The results of intestinal tissue staining with H&E are shown in Figure 5A. In rats in the MOD group, severe intestinal mucosal damage was observed, characterized by shortened and thinned villi, widened villi spacing, necrosis and collapse of intestinal epithelial cells, infiltration of inflammatory cells, gaps beneath the epithelium, oedema in the lamina propria and capillary bleeding. After GP intervention, the intestinal mucosa appeared healthier, with significantly reduced villous spacing, minor collapse of small intestinal epithelial cells and mild congestion and oedema beneath the epithelial cells. Furthermore, as shown in Figure 5B, compared with the rats in the CON group, the serum levels of IL-2, IL-6 and TNF-α were significantly elevated in rats in the MOD group, whereas the levels of IL-4, IgG and IgA were significantly reduced. High-dose GP was able to reverse these changes. These results confirmed our previous hypothesis that polysaccharides from glycyrrhiza are key active components involved in the immune regulation of the body.

### 3.4 GP-1 balanced the gut microbiota in immunocompromised rats

**Figure 6:**
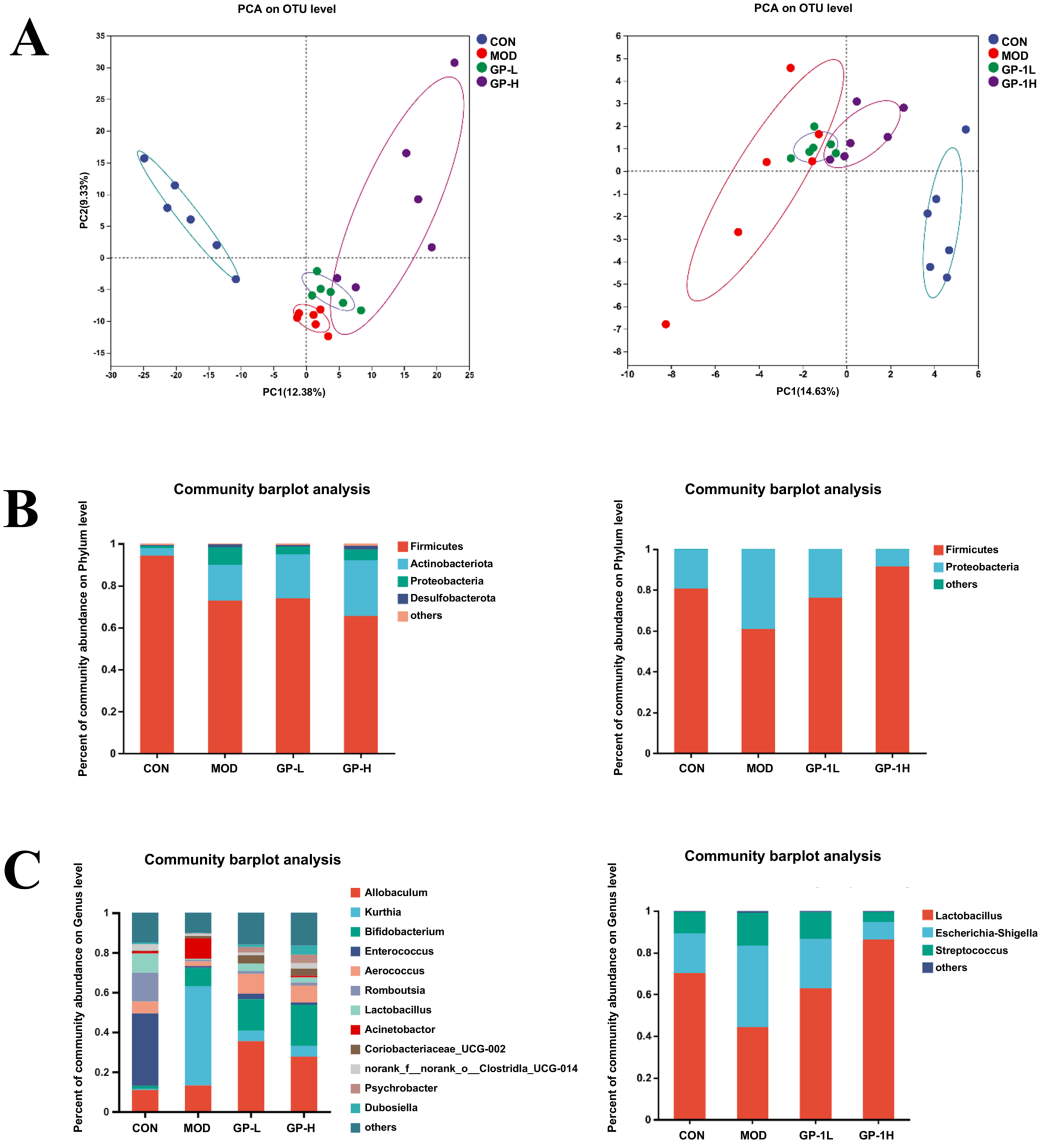
Overall gut microbiota composition *in vivo* and *in vitro* (*n* = 6). **(A)** Principal coordinate analysis (PCA) analysis at the operational taxonomic unit level for different groups. **(B)** Relative abundance at the phylum level; **(C)** Relative abundance at the genus level. CON, control group; MOD, model group; GP-L, low-dose GP group; GP-H, high-dose GP group; GP-1 L, low-dose GP-1 group; GP-1H, high-dose GP-1 group.

Because of the low yield and challenges associated with the large-scale preparation of homogeneous GP-1 in this study, crude GPs were used for *in vivo* experiments, whereas homogeneous GP-1 was used for *in vitro* experiments to investigate the regulatory effects of GPs on intestinal microbiota. Principal coordinate analysis (PCA) based on a phylogenetic assay demonstrated the relative clustering of the gut microbiota in each group (Figure 6A) and revealed significant separation between groups. The distinct segregation between the CON and MOD groups validated the efficacy of the immunocompromised model. Similarly, the clear differentiation between the low- and high-dose GP groups highlighted the significant effects of both doses on the intestinal flora of immunocompromised rats. Furthermore, the complete separation observed in the *in vitro* experiments with low- and high-dose GP-1 underscored the substantial alterations induced by the administration of both *in vitro* doses on the composition of the bacterial flora. At the phylum level (Figure 6B), both the *in vivo* and *in vitro* experimental results showed that the relative abundance of Firmicutes was significantly decreased in the MOD group, whereas that of Actinobacteriota was significantly increased compared with the CON group. Notably, in the *in vitro* fermentation experiment, GP-1 treatment reversed the changes in Firmicutes and Actinobacteriota, which was consistent with the effects observed with crude glycyrrhiza extract in our previous studies. However, in the *in vivo* experiment, following intervention with crude GPs, the relative abundance of Firmicutes was further decreased, whereas that of Actinobacteriota continued to increase. To investigate the cause of this discrepancy, we analysed differences in the composition of the gut microbiota at the genus level. We observed that GP treatment led to a significant reversal in the relative abundance of Kurthia, a genus in the Firmicutes phylum and a noticeable increase in the relative abundance of Bifidobacterium, a probiotic belonging to the Actinobacteriota phylum. These shifts are likely key factors behind abnormal alterations in gut microbiota composition at the phylum level. Notably, the composition of the gut microbiota was significantly simpler in the *in vivo* experiments than in the *in vitro* experiments. In the *in vitro* experiments, the gut microbiota in each group was mainly composed of Lactobacillus, Escherichia-Shigella and Streptococcus, which may be due to the unsuitability of *in vitro* conditions for the growth and proliferation of most microbial communities. After the GP-1 intervention, the relative abundance of the probiotic Lactobacillus significantly increased, whereas that of the harmful bacteria Escherichia-Shigella decreased (Figure 6C).

**Figure 7:**
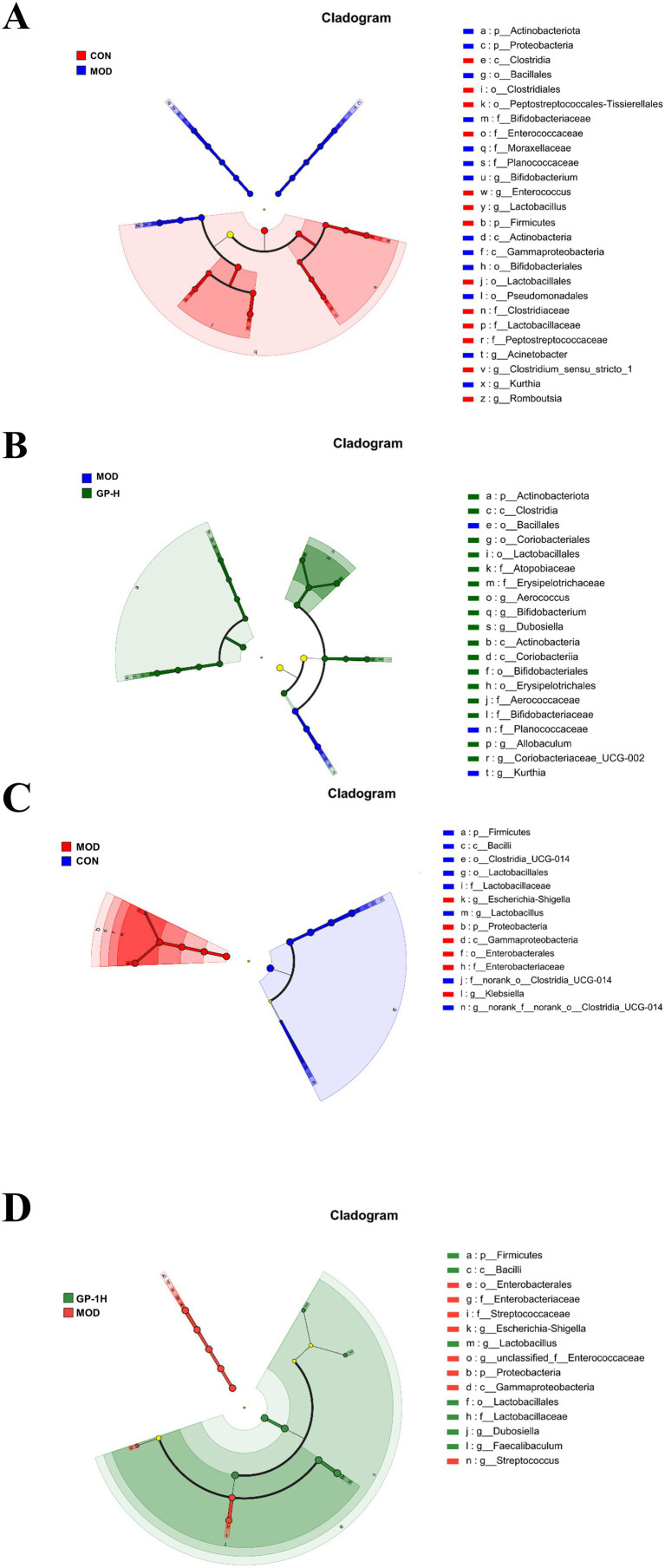
Linear discriminant analysis effect size (LEfSe) analysis of intestinal flora evolution *in vivo* and *in vitro* (*n* = 6). **(A)** Intestinal flora comparison between the *in vivo* CON and MOD groups; **(B)** Intestinal flora comparison between the *in vivo* MOD and GP-H groups; **(C)** Intestinal flora comparison between *in vitro* control and MOD groups. **(D)** Intestinal flora comparison between the *in vitro* MOD and GP-1H groups. CON, control group; MOD, model group; GP-H, high-dose GP group; GP-1H, high-dose GP-1 group (LDA > 4, *p* < 0.05).

**Figure 8:**
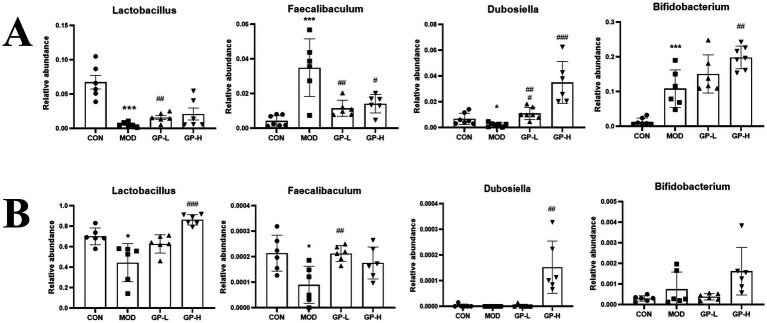
Comparative analysis of representative intestinal bacteria (*n* = 6). **(A)** Representative intestinal flora *in vivo*. **(B)** Representative intestinal flora *in vitro*. CON, control group; MOD, model group; GP-L, low-dose GP group; GP-H, high-dose GP group; GP-1 L, low-dose GP-1 group; GP-1H, high-dose GP-1 group. The values are expressed as means ± SD. The repeated measures data were analyzed using one-way ANOVA and Sidak *post hoc* test (vs. CON, *p < 0.05, **p < 0.01, ***p < 0.001; vs. MOD, #*p* < 0.05, ##*p* < 0.01, ###*p* < 0.001).

Because of the complexity of the gut microbiota, we conducted linear discriminant analysis Effect Size (LEfSe) analysis (LDA > 4.0, *p* < 0.05) on the rats in the CON, MOD and high-dose GP groups (GP-H and GP-1H) to further investigate the *in vivo* and *in vitro* fermentation characteristics of GPs. The results indicated that in the *in vivo* experiment, following a controlled diet and excessive swimming, the levels of three genera (Acinetobacter, Bifidobacterium and Kurthia) significantly increased, whereas those of four communities (Romboutsia, Clostridium_sen- su_stricto_1, Enterococcus and Lactobacillus) significantly decreased. After the GP intervention, at the genus level, a significant reversal was observed in the abnormal increase in Kurthia, along with an increase in the relative abundance of Aerococcus, Allobaculum, Dubosiella, Coriobacteriaceae_UCG-002 and Bifidobacterium (Figures 7A,B). In the *in vitro* experiment, compared with the CON group, at the genus level, the relative abundance of Lactobacillus and norank_f__no- rank_o__Clostridia_UCG-014 in the MOD group significantly decreased, whereas that of Escherichia-Shigella and Klebsiella significantly increased. Following the GP-1 intervention, at the genus level, a significant decrease was observed in the relative abundance of Escherichia-Shigella, Streptococcus and unclassified_f__E- nterococcaceae, whereas a significant increase was observed in the relative abundance of Lactobacillus, Dubosiella and Faecalibaculum (Figures 7C,D). Notably, despite the significant differences in gut microbiota composition between the *in vivo* and *in vitro* experiments, the relative abundance of Dubosiella (42), an SCFA-producing bacterium, significantly increased in both cases following polysaccharide intervention, which was consistent with our previous findings following glycyrrhiza extract intervention (23). Furthermore, in the *in vitro* experiment, the three communities that significantly increased after homogeneous GP-1 intervention were SCFA-producing bacteria. Therefore, we focused on examining changes in the relative abundances of several SCFA-producing bacteria in both *in vivo* and *in vitro* experiments, as shown in Figure 8. Following polysaccharide intervention, a significant increase was observed in the relative abundance of Lactobacillus, Dubosiella, Faecalibaculum and Bifidobac- terium in both *in vivo* and *in vitro* fermentation experiments. In particular, Lactobacillus and Dubosiella exhibited results consistent with our previous studies on raw and roasted glycyrrhiza interventions, further demonstrating that GP is a key active component of glycyrrhiza.

**Figure 9:**
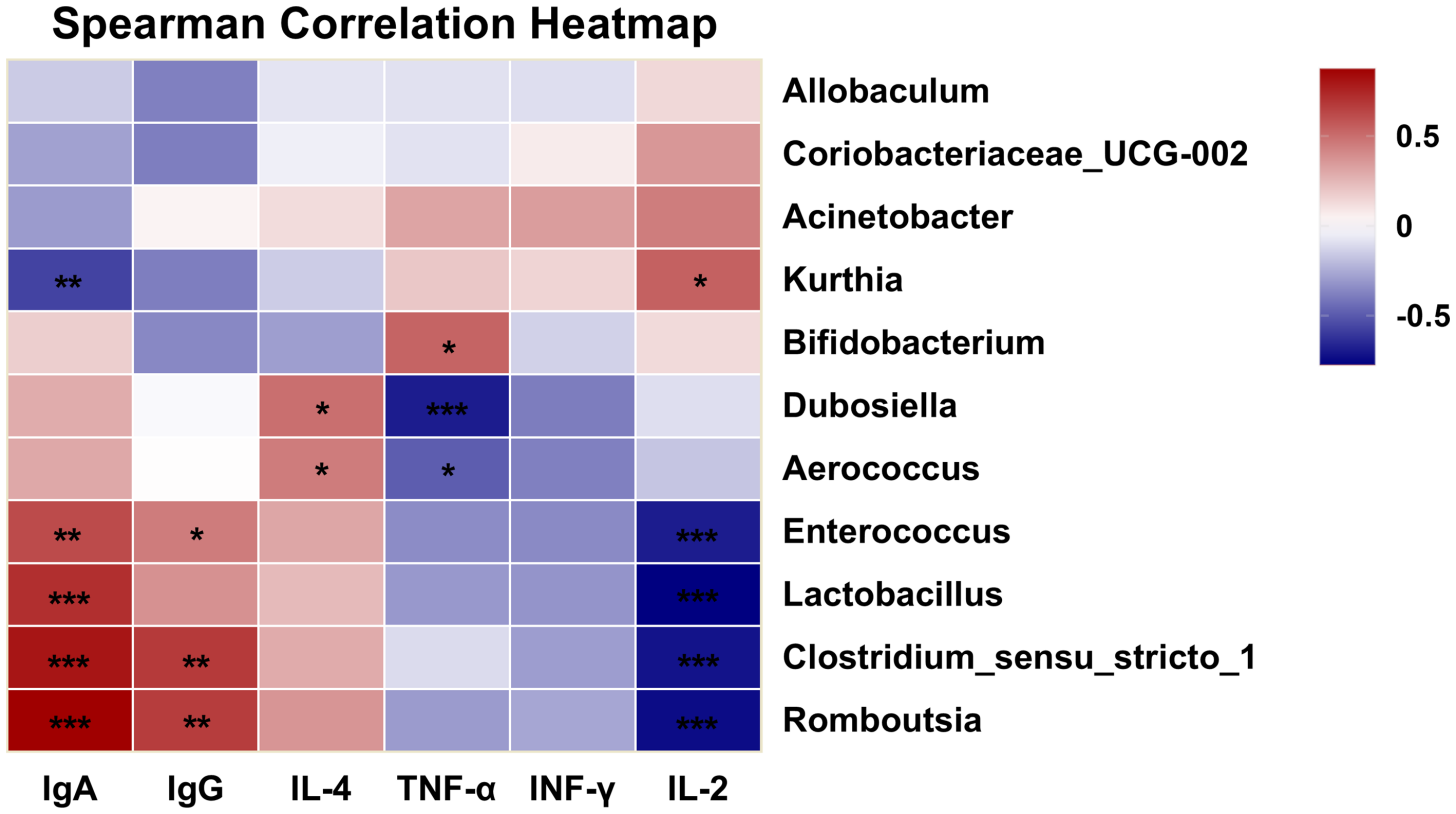
Correlation heatmap of immunity and gut microbiota at the genus level (*p < 0.05, **p < 0.01, ***p < 0.001).

To explore the potential relationship between gut microbiota composition and host immune responses, Spearman’s correlation analysis was performed between the differentially abundant genera and immune indices obtained from the *in vivo* experiments (Figure 9). In the present study, several bacterial genera showed statistically significant associations with immune parameters *in vivo*. For example, Lactobacillus displayed a positive association with IgA levels, whereas Dubosiella was negatively associated with TNF-α levels. These observations suggest that alterations in certain gut microorganisms may coexist with changes in mucosal immunity or inflammatory status; however, the current correlation-based analysis cannot determine whether these microbiota shifts play a direct role in modulating immune function, nor whether both changes are driven by other host or environmental factors. Future studies employing causal inference approaches—such as gnotobiotic animal models, targeted microbial transplantation, or mechanistic *in vitro* assays—will be required to verify and elucidate the biological significance of these associations.

*Lactobacilli* are common probiotics and beneficial commensal microorganisms in the human body, primarily residing in the gastrointestinal tract and female urogenital system as part of the symbiotic microbiota. They play a crucial role in preventing chronic diseases such as inflammatory bowel disease and are essential components of both the gut and vaginal microbiomes (43). Furthermore, most *Lactobacillus* species, as probiotic microorganisms, produce enzymes with antibiotic, anticancer and immunomodulatory properties (44).

*Dubosiella* shows promise as a probiotic, with early studies indicating its potential for treating obesity, hypertension and liver diseases (45–47). Furthermore, various studies have indicated that polysaccharide interventions can significantly enhance the abundance of *Dubosiella* (43, 48, 49). Similarly, this study yielded comparable results. In summary, GPs play a crucial role in regulating the balance of the gut microbiota and increasing the relative abundance of certain probiotics.

### 3.5 GP intervention increased SCFA content after *in vivo* and *in vitro* fermentation

**Figure 10:**
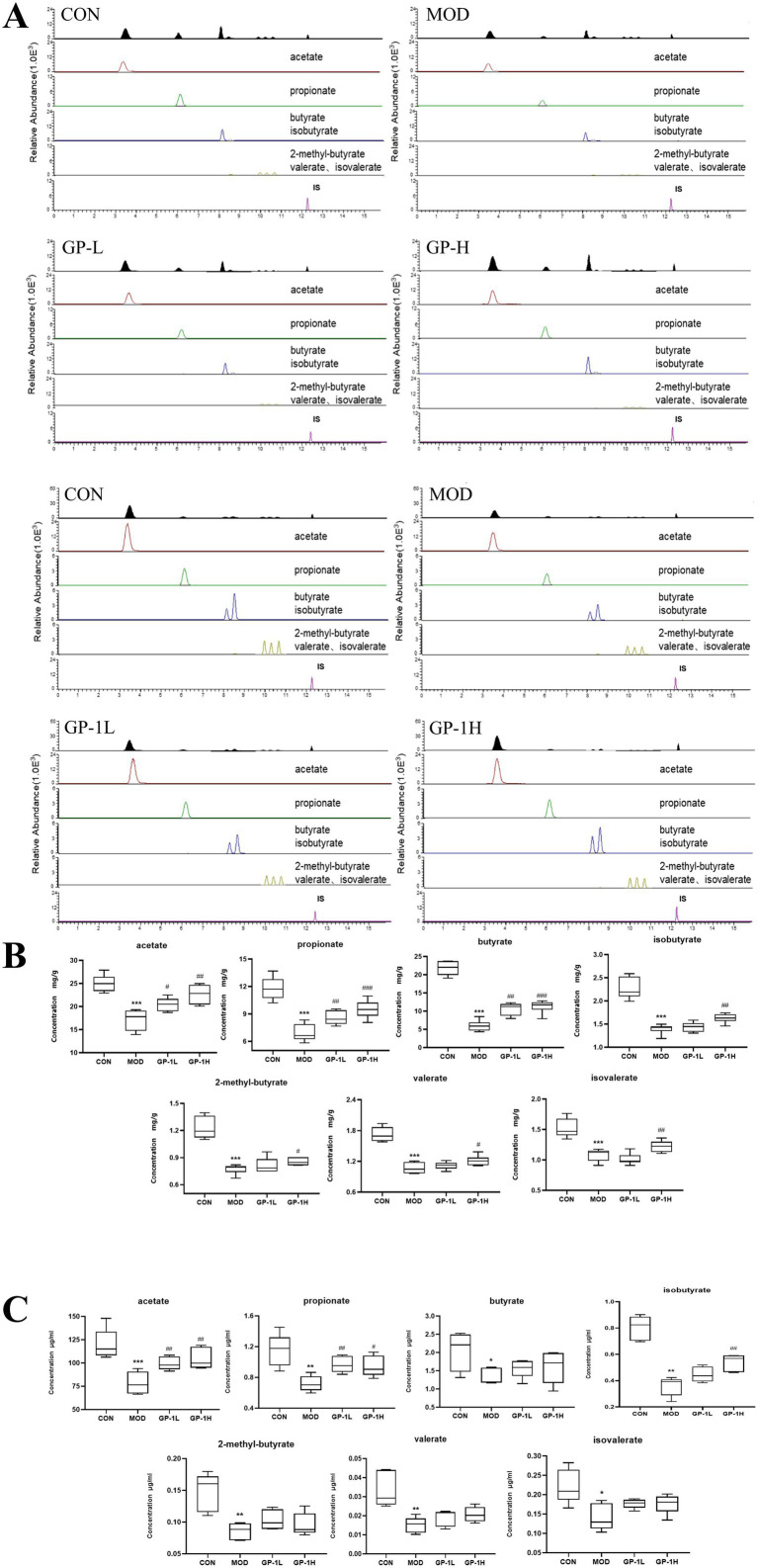
Levels of short-chain fatty acids (SCFAs) *in vivo* and *in vitro* (*n* = 6). **(A)** Representative chromatogram for SCFA quantification. **(B)** SCFA levels *in vivo*. **(C)** SCFA levels *in vitro*. CON: control group, MOD: model group, GP-L, low-dose GP group; GP-H, high-dose GP group; GP-1 L, low-dose GP-1 group; GP-1H, high-dose GP-1 group. The values are expressed as means ± SD. The repeated measures data were analyzed using one-way ANOVA and Sidak post hoc test (vs. CON, *p < 0.05, **p < 0.01, ***p < 0.001; vs. MOD, #*p* < 0.05, ##*p* < 0.01).

SCFAs are key bacterial metabolites involved in the intestinal immune response and important for regulating the permeability of the intestinal mucosal barrier (50). Given the observed increase in the relative abundance of beneficial SCFA-producing bacteria in the body, we subsequently determined the levels of acetate, propionate, butyrate, 2-methylbutyrate, isobutyrate, valerate and isovalerate in samples from *in vivo* and *in vitro* experiments (Figure 10A). As shown in Figure 10B, the concentration of SCFAs was compared between the immunodeficient MOD and CON groups (*p* < 0.001), revealing an imbalance and impairment in the metabolic activity of gut microbiota. After treatment with GP in the *in vivo* experiment, the levels of various SCFAs significantly increased, particularly acetate, propionate and butyrate. In the *in vitro* experiment (Figure 10C), after GP-1 treatment, only the levels of acetate, propionate and isovalerate significantly increased. This difference may be attributed to material differences between the *in vivo* and *in vitro* experiments, as well as significant differences in gut microbiota composition owing to environmental differences between the two experimental conditions.

Acetate is an SCFA that can be absorbed into the bloodstream primarily through the fermentation of carbohydrates by gut bacteria. Once in the blood, it is transported to the liver, where it is metabolized and used in various processes, including lipid and cholesterol synthesis. Acetate is an important energy source in tissues and plays a significant role in the regulation of metabolism. Additionally, it has implications for immune function by promoting the differentiation of regulatory T cells via the inhibition of the histone deacetylase enzyme HDAC9, which influences gene expression and immune responses (20). Propionate, which is absorbed by the colon, can be used for gluconeogenesis, reducing cholesterol synthesis and activating GPR41, which lowers IL-4, IL-5 and IL-13 levels in allergic airway inflammation in mice, quickly alleviating the inflammatory response (51). These findings indicate that the immune-modulating effects of GPs are closely associated with SCFAs.

## 4 Conclusion

In this study, we isolated a new neutral polysaccharide (GP-1) from *G. uralensis* Fisch. GP-1 is a glucan with a molecular weight of 7.6 kDa, and its main chain is connected via → 4)-α-D-Glc*p*-(1 → linkages. The β-D-Glc*p*-(1 → 6)-α-D-Glc*p*-(1 → branches are located at the C-6 position of 1,4,6-Glc, whereas the β-D-Glc*p*-(1 → branches are located at the C-3 position of 1,3,4-Glc. GP-1 can be degraded and consumed by the intestinal flora, significantly altering the composition of the intestinal flora and increasing the abundance of beneficial bacteria. In addition, GP-1 promotes the production of SCFAs, particularly acetic and propionic acids. In summary, GP-1 exhibits good probiotic properties; however, the mechanisms underlying its regulatory effects on intestinal bacteria and metabolic pathways require further investigation. Future research should focus on enriching GP-2 and investigating the biological activities and structure–activity relationships of these polysaccharides.

## Data Availability

The original contributions presented in the study are included in the article/Supplementary material, further inquiries can be directed to the corresponding author.
