# Supplementary material for: Structural characteristics of a neutral Glycyrrhiza uralensis polysaccharide and its fermentation properties on the gut microbiota of immunocompromised rats in vivo and vitro
Source: Front Nutr. 2025 Oct 7;12:1651015. doi: 10.3389/fnut.2025.1651015 (PMC12537741; doi:10.3389/fnut.2025.1651015)
Supplement: Supplementary file 1 [file Supplementary_file_1.docx]

**Supplementary material**

Structural characteristics of a neutral *Glycyrrhiza uralensis* polysaccharide and its fermentation properties on the gut microbiota of immunocompromised rats *in* *vivo* and *vitro*

Jie Sun ^a,b, c^, Yi-Xuan Wu ^a,b, c^, Xin-Li Li ^a,b, c^, Wen-Jie Xie ^a,b, c^ ,Qing-Ping Xiong ^e^ , Chang-Xing Jiang ^e^, Shu-Wan Tang ^a^, Guo-Ping Peng ^a,b, c,d^, Yun-Feng Zheng ^a,b, c,d, *^

**Affiliation**

*^a^ Department of Pharmacy, Nanjing University of Chinese Medicine, Nanjing 210046，China*

*^b^ National Key Laboratory on Technologies for Chinese Medicine Pharmaceutical Process Control and Intelligent Manufacture, Nanjing, 211100, China*

*^c^ Jiangsu Province Engineering Research Center of Classical Prescription, Nanjing University of Chinese Medicine, Nanjing 210023, China*

*^d^ Jiangsu Collaborative Innovation Center of Chinese Medicinal Resources Industrialization, Nanjing University of Chinese Medicine, Nanjing 210023, China*

*e Jiangsu Key Laboratory of Regional Resource Exploitation and Medicinal Research,* *Huaiyin Institute of Technology, Huai’an* *223003, Jiangsu, PR China*

Jie Sun：sunjiexueyong@163.com

Yi-Xuan Wu：wyx09272021@163.com

Xin-Li Li： lixinli2023@163.com

Wen-Jie Xie：xwenj0107@163.com

Qing-Ping Xiong：qpxiong@gmail.com

Chang-Xing Jiang：j1c2x3@gmail.com

Shu-Wan Tang：tang_swan@163.com

Guo-Ping Peng：320787@njucm.edu.cn

***Correspondence**

Yun-Feng Zheng, PhD, Professor.

Tel: +86 25 86798186, Fax: +86-25-8679-8186

E-mail: zyunfeng@njucm.edu.cn

**S1 Contents of sugar, protein, and glycuronic acid assay**

- 1. **Determination of total sugar content of GP, GP-1**

Take 10 mg of anhydrous glucose control, weigh it precisely, put it in a 100 mL measuring flask, add appropriate amount of water to dissolve it, dilute it to the scale, shake well, that is, the standard solution. Precisely measure 0.2 mL, 0.4 mL, 0.6 mL, 0.8 mL, 1.0 mL of control solution, respectively, placed in a stoppered test tube, respectively, add water to make up to 2.0 mL, accurately add 5% phenol solution 1 mL, vibration, quickly and precisely add sulfuric acid 5 mL, vibration, placed for 10 minutes, placed in a 40 ℃ water bath insulation for 15 minutes, take out, quickly cooled to room temperature, with the corresponding reagent as a blank. Use the corresponding reagent as blank, determine according to the UV-visible spectrophotometry, measure the absorbance at 490 nm, take the absorbance as the vertical coordinate and the weight as the horizontal coordinate, and draw the standard curve.

Take GP, GP-110 mg, weigh it precisely, put it in a 100 mL measuring flask, add appropriate amount of water to dissolve it, dilute it to the scale, take 0.5mL of the sample solution, add 1.5mL of water, according to the preparation method of the standard curve, determine the absorbance, read the weight of the total sugar in the test solution according to the standard curve and calculate it.

- 1. **Determination of GP-1 protein content**

Take 1.5mL centrifuge tubes numbered and add the reagents sequentially according to the table below. Mix well, let it stand for 2 minutes and determine the absorbance at 595 nm. The standard curve was plotted with protein concentration (μg/mL) as the horizontal coordinate and absorbance value as the vertical coordinate.

Take GP-110 mg, weigh it precisely, put it in a 10mL measuring flask, add appropriate amount of water to dissolve it, dilute it to the scale, get the test solution. Take 10 μL of the test solution, add 990 μL of Caulmers Brilliant Blue G-250 solution, mix well, let it stand for 2 minutes, and then use the No. 0 tube of the standard curve as a reference to determine the absorbance at 595 nm, and then read the concentration of protein in the test solution according to the standard curve and calculate.

- 1. **Determination of GP-1 galacturonic acid**

Take 10 mg of galacturonic acid control product, weigh it precisely, put it in a 10mL volumetric flask, add appropriate amount of water to dissolve it, dilute it to the scale, and shake it well, i.e. it is the standard solution. Take the standard solution 0.1 mL, 0.2 mL, 0.3 mL, 0.4 mL, 0.5 mL and 0.6 mL in a 10mL volumetric flask, add water and then take 1.0mL of each of the above prepared solutions to a 20mL stoppered test tube, placed in an ice-water bath, add sodium tetraborate / sulfuric acid solution (0.478g of sodium tetraborate dissolved in 100 mL of concentrated sulfuric acid) 6mL, to be all the After adding, mix with vortex mixer, heat in boiling water bath for 5min, cool in ice water bath and add m-hydroxybiphenyl solution (0.15g m-hydroxybiphenyl dissolved in 5 mg/mL NaOH solution, and then volume to 100 mL) 100 μL with micro-sampling gun, mix and shake for 5min, and ultrasonicate to remove the air bubbles. The absorbance was measured at the maximum absorption wavelength with 1mL of distilled water as above. The absorbance was measured at the maximum absorption wavelength. The absorbance was taken as the vertical coordinate and the concentration as the horizontal coordinate, and the standard curve was plotted.

Take GP-110 mg, weigh it precisely, put it in 10 mL measuring flask, add appropriate amount of water to dissolve it, dilute it to the scale, take 1mL of the sample solution into a 20mL stoppered test tube, refer to the configuration method under the standard curve, determine the absorbance, and read the concentration of glucuronic acid in the test solution according to the standard curve and calculate it.

**S2 Monosaccharide composition analysis**

Take appropriate amount of rhamnose, galacturonic acid, glucose, galactose, arabinose control, precision weighing, add water to configure every 1 mL containing rhamnose 0.379 mg, galacturonic acid 0.323 mg, glucose 1.208 mg, galactose 0.437 mg, arabinose 0.312 mg of mixed control solution, that is, the control solution.

Take GP-110 mg, weigh precisely, put in 100 mL measuring flask, add water to dissolve, dilute to scale. Precision measure 2.0 mL in an anatomical flask, add trifluoroacetic acid to 2.0 mol/L, 100 ℃ hydrolysis 6 h, evaporation, repeatedly add methanol evaporation to neutral, the residue with water to dissolve and volume to 2.0 mL of water, that is, the GP-1 hydrolysis solution.

Hydrolyzed sample solution, control solution 100 μL, add 0.3 mol/L sodium hydroxide aqueous solution 100 μL, 0.5 mol-L-1 PMP methanol solution 200 μL, mixing, 70 ℃ water bath for 45 min, cooled to room temperature, add 0.3 mol/L hydrochloric acid solution 100 μL and 500 μL of water in turn, mixing. Add chloroform 1 mL extraction, remove the organic phase after sufficient shaking, repeat the extraction 3 times, the aqueous phase solution was filtered through 0.22 μm microporous membrane, the filtrate was taken, that is, the test solution.

Liquid chromatography analysis was performed using an Agilent 1100. Sample separation was achieved on a Hedra C18 column (4.6 × 250 mm, 5 μm) at a constant flow rate of 1.0 mL/min. The mobile phase (pH = 6.5) consisted of acetonitrile (A) and ammonium acetate buffer solution (B), and was eluted using a gradient of 15 %A - 25 %A from 0 to 65 min, with a detection wavelength of 254 nm and the injection volume set to 5 μL.

**S3 Measurement of SCFAs by chemical derivatization and UPLC-MS/MS**

40 mg of fecal samples homogenized in 0.5 mL 50% acetonitrile and centrifuged at 4,000 rpm for 10 minto separate particulates from supernatant containing SCFAs.

30 μL of fecal warm incubation solution was mixed with 70 μL of acetonitrile and centrifuged at 13000 rpm for 10 min.

40 μL of both supernatants was derviatived with 5μL IS, 20 μL 3-nitrophenylhydrazine (3-NPH) and 20 μL 1-ethyl-3-(3-dimethylaminopropyl) carbodiimide (EDC)-6% pyridine·solution at 40 °C for 30 min. Transfer the solution to a 1-mL volumetric flask and to make up to the mark with water, then mix. The solution was centrifuged at 18,000 rpm for 10 min, and the supernatant was transferred to an auto-sampler vial for UPLC-MS/MS analysis.

Liquid chromatographic analysis was performed using the DIONEX Ultimate 3000 UPLC system (Thermo Fisher Scientific). Sample separation was achieved on a BDS hypersil-C18 column (2.1 × 100 mm, 2.4 µm) with a constant flow rate of 0.3 mL/min at 50 °C. The mobile phase was composed of water (0.1% formic acid, A) and ACN (0.1% formic acid, B), using a gradient elution of 10% B at 0-3 min, 10-35% B at 3-10 min, 35-95 B at 10-12 min, 95% B at 12-14 min, 95-10% B at 14-14.5 min. The injection volume was set at 5.0 µL.

The measurement of 3-NPH derivatized SCFAs was performed on a triple quadrupole mass spectrometer (TSQ Vantage; Thermo Fisher Scientific Inc., San Jose, CA, USA). Using an electrospray ion source (ESI), the analyte was analyzed in a multi-reaction detection (MRM) mode under negative ion modes scanning. MS parameters were as follows: spray voltage, 3.2 kV; sheath gas/auxiliary gas, nitrogen; sheath gas pressure, 45bar; auxiliary gas pressure, 25bar; ion transfer capillary temperature, 300°C. Data analysis and processing were performed using Xcalibur software, v. 3.1.66.10.


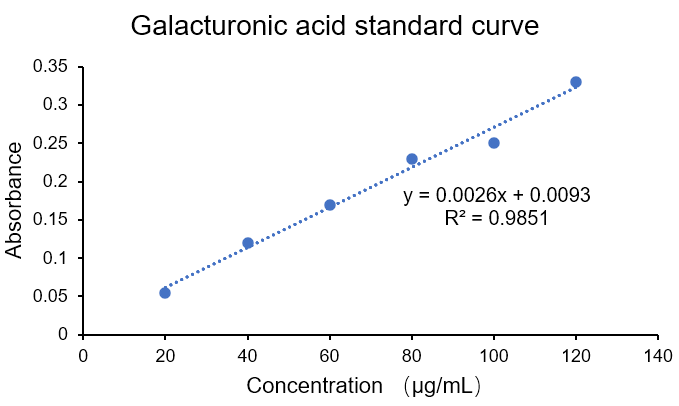

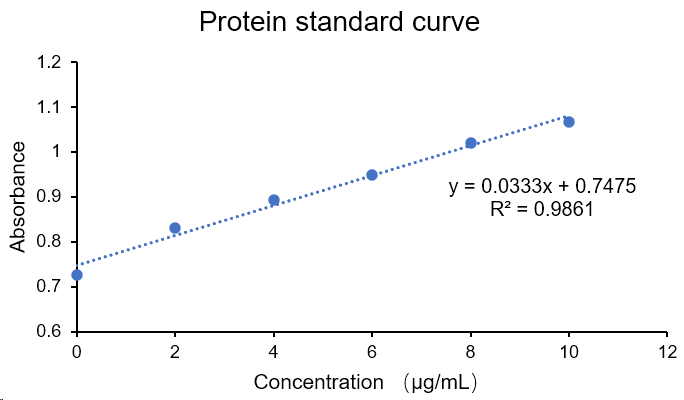

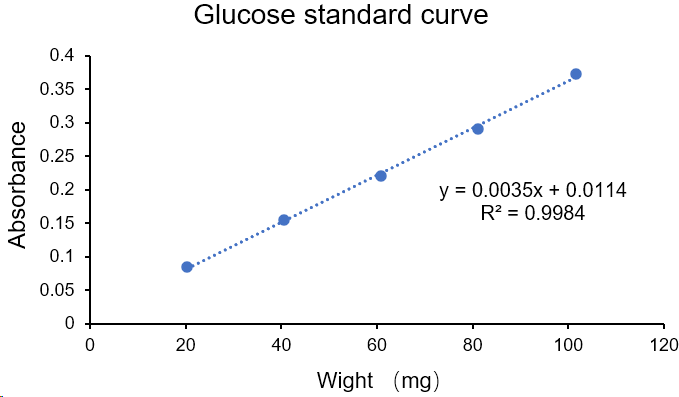


**A**

**C**

**B**

**Figure S1.** Standard Curve for Sugar, Protein, Glycuronic Acid Determination.

A: Glucose standard curve; B: Protein standard curve; C: Galacturonic acid standard curve.
